# Supplementary material for: The brown adipocyte protein CIDEA promotes lipid droplet fusion via a phosphatidic acid-binding amphipathic helix
Source: eLife. 2015 Nov 26;4:e07485. doi: 10.7554/eLife.07485 (PMC4755750; doi:10.7554/eLife.07485)
Supplement: Figure 6—source data 1. — DOI: http://dx.doi.org/10.7554/eLife.07485.017 [file elife-07485-fig6-data1.zip › Figure 6-source data 1.pdf]

SUPPORTING INFORMATION

Table S1. List of yeast strains used in this study.

Strain and Ref.	Genotype	Source
**Wild type (BY4742)** (1)	*MAT*α; *his3Δ1*, *leu2Δ0*, *lys2Δ0*, *ura3Δ0* carrying pRS316-CYC1p (*URA3 CEN6 CIDEA)*	
***dgk1*Δ** (2)	*dgk1:: KanMX, his3Δ1, leu2Δ0, lys3Δ0, ura3Δ0* carrying pRS316-CYC1p (*URA3 CEN6 CIDEA)*	M.L. Gaspar
***dgk1*Δ*pah1*Δ** (2, 3)	*dgk1:: KanMX pah1Δ::LEU2, his3Δ1, leu2Δ0, lys3Δ0, ura3Δ00* carrying pRS316-CYC1p (*URA3 CEN6 CIDEA)*	M.L. Gaspar
***cho2*Δ** (4)	*cho2:: KanMX his3Δ1, leu2Δ0, lys3Δ0, ura3Δ0* carrying pRS316-CYC1p (*URA3 CEN6 CIDEA)*	S.A. Henry
***pld1*Δ** (5)	*pld1:: HIS3, his3Δ1, leu2Δ0, ura3Δ0* carrying pRS316-CYC1p (*URA3 CEN6 CIDEA)*	S.A. Henry
***dga1*Δ*lro1*Δ*are1*Δ*are2*Δ** (6-8)	*dga1::Kan*MX, *lro1::Kan*MX*, are1::Kan*MX*, are2::Kan*MX*, his3Δ1*, *leu2Δ0*, *lys2Δ0*, *ura3Δ0* carrying pRS316-CYC1p (*URA3 CEN6 CIDEA)*	S.D. Kohlwein
***tgl3*Δ*tgl4*Δ*tgl5*Δ** (9, 10)	*tgl3::Kan*MX, *tgl4::Kan*MX*, tgl5::Kan*MX*, his3Δ1*, *leu2Δ0*, *lys2Δ0*, *ura3Δ0* carrying pRS316-CYC1p (*URA3 CEN6 CIDEA)*	S.D. Kohlwein
PAH1-7A (11, 12)	*MAT*α; *his3Δ1*, *leu2Δ0*, *lys2Δ0*, *ura3Δ0* carrying pRS415- *PAH1*$^{S110A/S114A/S168A/S602A/T723A/S744A/S748A}$ (*LEU2 CEN6 PAH1-7A*) + pRS316-CYC1p (*URA3 CEN6 CIDEA)*	This study

1. Brachmann CB, Davies A, Cost GJ, Caputo E, Li J, Hieter P, et al. Designer deletion strains derived from Saccharomyces cerevisiae S288C: a useful set of strains and plasmids for PCR-mediated gene disruption and other applications. Yeast. 1998;14(2):115-32.
2. Han GS, O'Hara L, Carman GM, Siniossoglou S. An unconventional diacylglycerol kinase that regulates phospholipid synthesis and nuclear membrane growth. J Biol Chem. 2008;283(29):20433-42.
3. Santos-Rosa H, Leung J, Grimsey N, Peak-Chew S, Siniossoglou S. The yeast lipin Smp2 couples phospholipid biosynthesis to nuclear membrane growth. EMBO J. 2005;24(11):1931-41.
4. Summers EF, Letts VA, McGraw P, Henry SA. Saccharomyces cerevisiae cho2 mutants are deficient in phospholipid methylation and cross-pathway regulation of inositol synthesis. Genetics. 1988;120(4):909-22.
5. Rose K, Rudge SA, Frohman MA, Morris AJ, Engebrecht J. Phospholipase D signaling is essential for meiosis. Proc Natl Acad Sci U S A. 1995;92(26):12151-5.
6. Oelkers P, Tinkelenberg A, Erdeniz N, Cromley D, Billheimer JT, Sturley SL. A lecithin cholesterol acyltransferase-like gene mediates diacylglycerol esterification in yeast. J Biol Chem. 2000;275(21):15609-12.
7. Oelkers P, Cromley D, Padamsee M, Billheimer JT, Sturley SL. The DGA1 gene determines a second triglyceride synthetic pathway in yeast. J Biol Chem. 2002;277(11):8877-81.
8. Sandager L, Gustavsson MH, Stahl U, Dahlqvist A, Wiberg E, Banas A, et al. Storage lipid synthesis is non-essential in yeast. J Biol Chem. 2002;277(8):6478-82.
9. Athenstaedt K, Daum G. Tgl4p and Tgl5p, two triacylglycerol lipases of the yeast Saccharomyces cerevisiae are localized to lipid particles. J Biol Chem. 2005;280(45):37301-9.
10. Kurat CF, Wolinski H, Petschnigg J, Kaluarachchi S, Andrews B, Natter K, et al. Cdk1/Cdc28-dependent activation of the major triacylglycerol lipase Tgl4 in yeast links lipolysis to cell-cycle progression. Mol Cell. 2009;33(1):53-63.
11. Choi HS, Su WM, Morgan JM, Han GS, Xu Z, Karanasios E, et al. Phosphorylation of phosphatidate phosphatase regulates its membrane association and physiological functions in Saccharomyces cerevisiae: identification of SER(602), THR(723), AND SER(744) as the sites phosphorylated by CDC28 (CDK1)-encoded cyclin-dependent kinase. J Biol Chem. 2010;286(2):1486-98.
12. Choi HS, Su WM, Han GS, Plote D, Xu Z, Carman GM. Pho85p-Pho80p phosphorylation of yeast Pah1p phosphatidate phosphatase regulates its activity, location, abundance, and function in lipid metabolism. J Biol Chem. 2012;287(14):11290-301.
